# Learning curves, safety, and experiences of a tertiary surgical center in the introduction of robotic-assisted surgery in gynecologic oncology

**DOI:** 10.1007/s00404-026-08349-8

**Published:** 2026-02-10

**Authors:** Lisa Jung, Florin-Andrei Taran, Sarah Huwer, Benedikt Kurz, Maximilian Klar, Angeline Favre-Inhofer, Ingolf Juhasz-Böss

**Affiliations:** 1 Department of Obstetrics and Gynecology at the Medical Center, University of Freiburg, Hugstetterstr. 55, 79106 Freiburg, Germany; 2 Department of Gynecology and Gynecologic Oncology, Center of Integrated Oncology Aachen Bonn Cologne Düsseldorf, Medical Faculty and University Clinic of Cologne, University of Cologne, Cologne, Germany; 3 Women’s Clinic, Cantonal Hospital Aarau, Aarau, Switzerland

**Keywords:** Robotic-assisted surgery, DaVinci, Gynecologic oncology, Learning curve, Safety

## Abstract

**Background:**

The dynamic development towards robotic-assisted surgery particularly affects operative gynecology. The analysis of operative data from robotic-assisted procedures since the first application at a surgical center provides valuable insights into the introduction phase and integration of the DaVinci system into routine clinical operations, as well as their impact on patient care. The aim of this work was to specifically examine the learning curve progression and to present the trend of the professionalization process in implementing the methodology in gynecologic oncology.

**Materials and methods:**

A retrospective data analysis was conducted of the first *n* = 107 patients who underwent surgery for a gynecological malignancy with the DaVinci surgical system at the University Medical Center Freiburg between 2020 and 2022. Classic operative parameters were evaluated, including preparation time, skin-to-skin time, console time, and the resulting learning curves of the surgeons and the operative team (including CUSUM analysis and linear regression models). Additionally, perioperative patient characteristics were recorded (e.g., blood loss, length of hospitalization, conversion rate).

**Results:**

The average operative preparation time is 26.11 ± 8.13 min. The maximum value (CUSUM peak) is at approximately 20 performed procedures, indicating that the processes of operative preparation were mastered after this number of operations. The average skin-to-skin time is 172.84 ± 71.68 min (range 43–387 min), whereby after an initial reduction in skin-to-skin time within the first 30 cases, there was a slight increase in the further course with renewed reduction from approximately 65 procedures. The average console time for all tumor entities is 131.98 ± 63.74 min; for the most common operative indication (endometrial cancer, *n* = 61), it is 109.89 ± 52.04 min (range 48–221 min). In the surgeons' learning curves, the two surgeons with the most procedures show a CUSUM peak after 11 and 22 procedures, respectively. The average length of stay is 5.00 days (± 2.30). A total of two conversions occurred (conversion rate = 1.9%).

**Discussion:**

Upon evaluation of the first *n* = 107 DaVinci operations, rapid learnability of robotic-assisted operations was demonstrated. The conversion rate was low at 1.9%. A positive effect on the learning curve of individual surgeons was evident after approximately 20 procedures. Both the preparation time and the skin-to-skin time could be rapidly reduced, so that integration into routine clinical operations was possible without problems.

## What does this study add to the clinical work

**Table:** 

Robot-assisted surgery in gynecological oncology can be safely and efficiently integrated into clinical practice. It has been shown that after approximately 10 to 20 performances of the same procedure, a significant increase in efficiency is achieved, and patient safety remains ensured throughout the learning phase. The combination of rapid learning curve, low conversion rates, and oncological outcomes comparable to conventional methods makes robot-assisted surgery a viable option for routine use in gynecological oncology.	

## Introduction

One of the most significant innovations in the surgical treatment of patients is robotic-assisted surgery. Surgical robotics, controlled by a surgeon, enables a new level of precision, gentler patient care, and the use of technical assistance systems. One of the first and most widely used robotic-assisted surgical systems is the da Vinci robotic system.

The applications of the da Vinci Surgical System are very diverse, which is why the system is used in a wide variety of surgical disciplines. In gynecology, it is primarily used for hysterectomy in cases of benign and malignant conditions.

Gynecological oncology is undergoing a remarkable transformation, which has gained further momentum since the approval of the da Vinci Surgical System and the application of robotic-assisted minimally invasive techniques. The steady increase in robotic-assisted surgeries in hospitals demonstrates that robotic surgery will play an increasingly important role in public healthcare in the coming years.

Current studies on operating times with the da Vinci Surgical System compared with conventional laparoscopy show inconsistent results. Several studies indicate longer operating times compared to conventional laparoscopy, citing robotic setup and dismantling as key time factors [1–3]. However, if the team is well-coordinated, the surgeon is experienced, and the procedures are well-structured, the operating time is comparable after the initial setup phase [4]. Furthermore, the results of Shashoua et al. (2009) [5] show that while the overall operating time is longer for robotic-assisted surgery, this depends on other co-factors, such as BMI.

Therefore, no definitive statement can yet be made about the time factor in robotic-assisted surgery, and further study data are needed.

However, implementing changes and innovations in a tightly scheduled, routine operating room initially presents many hospitals with significant challenges. Establishing a new system such as the da Vinci Surgical System is a change that, especially at the beginning, entails a substantial adjustment to the work environment, workflows, and task allocation [6]. The introduction of the da Vinci Surgical System at the Department of Gynecology at the University Hospital of Freiburg in February 2020 has significantly influenced the surgical care of patients in recent years.

Following the implementation of the new technology in our own cohort of benign total hysterectomies, we were able to demonstrate that both the preparation time and the incision-to-suture time were rapidly reduced, making integration into routine clinical practice seamless. The conversion rate was very low, at 0.8%, and a positive effect on the learning curve of individual surgeons was shown after approximately 20 benign hysterectomies [7].

This study presents, for the first time, both the surgeon's and the team's learning curves using CUSUM analysis. With a conversion rate of 1.9%, the Freiburg dataset is significantly lower than the rates reported in the literature for robotic gynecologic oncology surgery. Current studies report conversion rates of 2.2–7% for robotic-assisted procedures, with the conversion rate typically being higher during the implementation phase [8].

The aim of this study is to analyze surgical data from the first robotic-assisted gynecologic oncology procedures performed at the University Hospital of Freiburg. Based on this data, the implementation of robotic-assisted surgery, along with its advantages and disadvantages in patient care, will be evaluated. This includes analyzing the procedures performed, operating times, surgeons' learning curves, hemoglobin loss, patient length of stay, and the conversion rate. Sharing experiences and data from a successful implementation provides an important foundation for other hospitals to better overcome initial hurdles and advance the development of advanced technologies and applications.

## Methods

This study is a retrospective data analysis of the first robotic-assisted surgical procedures performed in the Department of Gynecology at the University Hospital of Freiburg. The analysis includes data from all patients who underwent surgery using the da Vinci Surgical System (Intuitive; MultiPort X system) between February 2020 and June 2022. These data were obtained from the electronic patient records and the hospital information system (HIS). The following parameters were collected: age, weight, height, BMI, length of stay, surgeon's name, diagnosis(es), the procedure(s) performed, and any special circumstances during the operation.

All surgical indications for gynecological malignancies were included in the analysis: endometrial cancer, cervical cancer, ovarian cancer, vulvar cancer, vaginal cancer, and uterine sarcoma. This study did not consider benign surgical indications such as uterine myomatosis, adenomyosis uteri, transgender surgery (female to male), bleeding disorders, prophylactic surgeries due to familial carcinoma risk, cervical intraepithelial neoplasia (CIN I–III).

### Operating times

In addition to the date of the operation, the following operating times were evaluated: start of preparation time, end of preparation time, patient in the operating room, start of surgical procedures, time of incision, start of console time, end of console time, and time of suturing completion.

The operating times are calculated based on the times documented by the operating room staff. The preparation time is calculated using the documented times for "patient in the operating room" and "incision." The skin-to-skin times are derived from the documented times for "incision" (skin incision; a uterine manipulator may have been applied beforehand) and "suturing completion" (skin suture). Console time is calculated using the documented times for "start of console time" (surgeon sits at the console and begins the first procedure) and "end of console time" (surgeon leaves the console).

### Learning curves

To create the learning curves, the procedures were assigned to individual surgeons. The chronological order of the procedures was always maintained for each surgeon. To analyze the most homogeneous comparison groups possible, the procedures were further subdivided according to the surgical indication and the type of procedure performed.

To evaluate the learning curves of the individual surgeons, the console times were graphically represented in a diagram and overlaid with a polynomial trend line. To specifically examine the learning curve progression and better illustrate the trend of the professionalization process during learning, a cumulative summation (CUSUM) analysis was also performed. This is an analysis method for measurement parameters with incremental changes, primarily used in business for process control and quality assurance. For this, the difference between the operating time and the corresponding mean value of the numerical series is calculated. The CUSUM values for the individual data series were then calculated by summing the respective preceding CUSUM value (for the first value = 0) with the difference between the operating time and the mean.

This procedure was performed for the entire data set in each case. The CUSUM diagram thus represents the cumulative sums of the deviations of the individual sample values from the mean (the target value), meaning that even small deviations lead to steadily increasing or decreasing cumulative deviation values. The graphical representation of the CUSUM analysis has the advantage of allowing an estimation, based on the CUSUM peak of the usually inverted parabolic curve, of how long it takes for the operator to master the learning curve [8]. This point in the learning process is often described in theory as the ability to perform the newly acquired task quickly and safely.

Microsoft^®^ Excel^®^ 2021 MSO (version 2409 build 16.0.18025.20030) was used for the statistical analysis and graphical representation of the data.

The study was approved by the Ethics Committee of the University Hospital of Freiburg (application number: 23-1501-S1-retro). In accordance with the submission guidelines and recommendations of the International Committee of Medical Journal Editors (ICMJE) and the Declaration of Helsinki, the manuscript explicitly confirms that the study was conducted according to the ethical principles of the responsible ethics committee and to international standards. Written informed consent was obtained from all study participants. Compliance with these ethical requirements was reviewed and approved by the responsible ethics committee of the University Hospital of Freiburg. The privacy and protection of the participants' personal data were ensured. The study was not funded. Clinical trial number: not applicable.

## Results

Between February 2020 and June 2022, a total of *n* = 107 patients underwent surgery for gynecological malignancy using the da Vinci Multiport X surgical robot. Patient characteristics are summarized in Table 1. Since the learning curves are limited to the subgroup of patients with endometrial cancer, Table 2 shows more detail.

**Table 1:** Characteristics of the patients

Mean age	57,1 (range 23–83)
BMI	30,5 (range 17,5–55,7)
ASA (I-VI)	2,2 ± 0,61
Hospitalization (days, average)	5 (range 1–17)
Uterine weight (g)	173 ± 168,2 (range 53–447)
Tumor entity (*n*)	
Endometrial cancer	61
Cervical cancer	34
BOT	5
Ovarian cancer	3
Vulvar cancer	2
Vaginal cancer	1
Uterine sarcoma	1

**Table 2:** Characteristics of the patients with endometrial cancer

Endometrial cancer *n* = 61	
Intraoperative adhesions	59,0%
SLNE	90,2%
Pelvic LND	3,3%
Pelvic and para-aortic LND	6,6%
Omenetectomy	3,3%
Colpectomy	4,9%
Pre-Operations	77,0%
Blood-loss (ml)	84,6 ± 151,8 (range10–1000)
BMI	33,5 ± 9,0 (range 18,4–55,7)

In the subgroup of patients with endometrial cancer, intraoperative adhesions were observed in 59.0%. Sentinel lymph node biopsy was performed in 90.2% of cases, while pelvic and pelvic-para-aortic lymphadenectomy were performed in 3.3% and 6.6% of cases, respectively. The median intraoperative blood loss was 50 ml (IQR 100–20), and the median body mass index was 33.5 kg/m^2^ (IQR 27.0–39.8).

A multivariable linear regression analysis was performed to evaluate the influence of surgical experience and procedural complexity on intraoperative blood loss. The model included the consecutive case number, sentinel lymph node dissection, complete lymphadenectomy, intraoperative adhesions, and a history of prior surgery as independent variables. The overall model was not significant (*R*^2^ = 0.021, *p* = 0.954).

The consecutive case number, representing surgical experience, showed no significant association with blood loss (*β* = −0.12 ml per case, *p* = 0.942). None of the additional covariates were significantly associated with blood loss.

### Surgical preparation

**Fig. 1:**
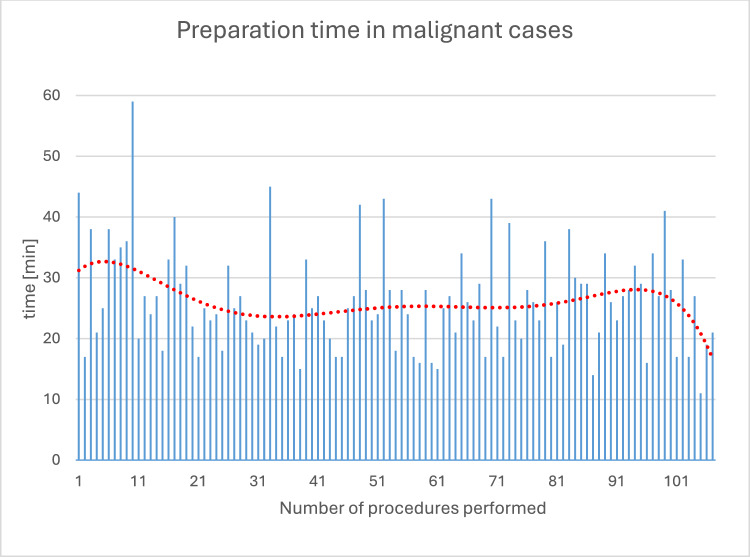
Preparation time in the entire cohort. Preparation time decreases with an increasing number of procedures performed. The red trend line (polynomial grade 5) shows that a significant reduction in preparation time was achieved within the first 30 procedures

**Fig. 2:**
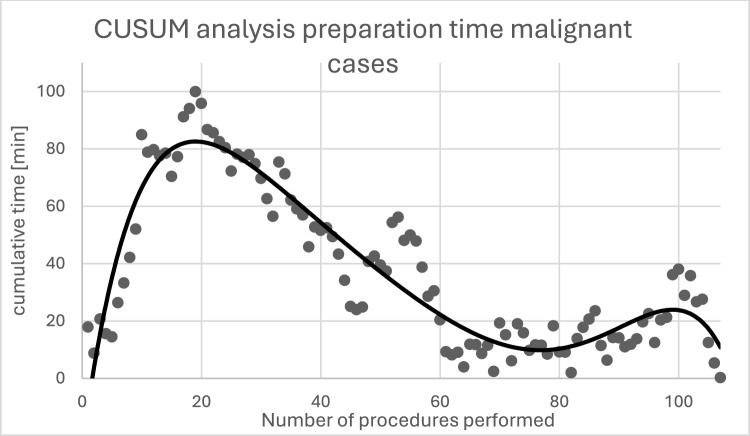
Shows the CUSUM values for preparation times. The CUSUM peak occurs after approximately 20 procedures have been performed

The duration of the surgical preparation time, defined as the time from "patient in the operating room" to "incision," averages 26.11 min (± 8,13) (Fig. 1). Figure 2 shows the analysis of the data using CUSUM analysis, which allows for a more precise estimation of when a routine is established in the surgical preparation process. The maximum value (CUSUM peak) of the inverse parabolic curve at approximately 20 procedures indicates that the surgical preparation processes were mastered after this number of operations.

### Skin-to-skin time

**Fig. 3:**
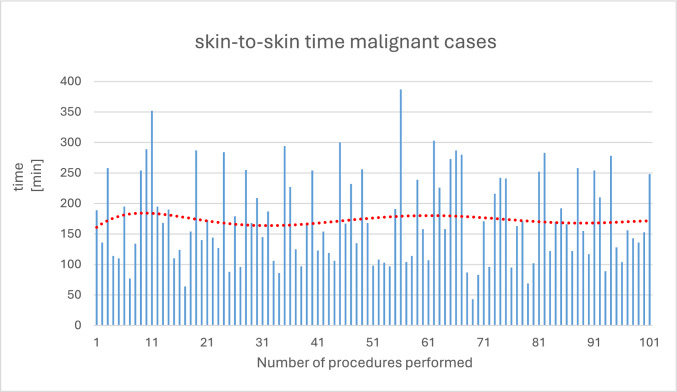
Shows skin-to-skin time in the entire cohort. The trend line in red (polynomial degree 6; *R*^2^ = 0.03) indicates a reduction in skin-to-skin time for the first 30 procedures. Subsequently, the skin-to-skin time increases again, before decreasing continuously thereafter

Patients with malignant surgical indications have an average skin-to-skin time of 172.84 min (± 71.68). The shortest skin-to-skin time for a patient is 43 min (endometrial cancer). The longest skin-to-skin time is 387 min for a patient with cervical carcinoma. The skin-to-skin times for individual operations in the entire cohort are shown in Fig. 3. The trend line in red indicates that after an initial reduction in skin-to-skin time within the first 30 cases, there is an increase thereafter. Following this slight increase, the skin-to-skin time decreases again from about 65 procedures onwards.

**Fig. 4:**
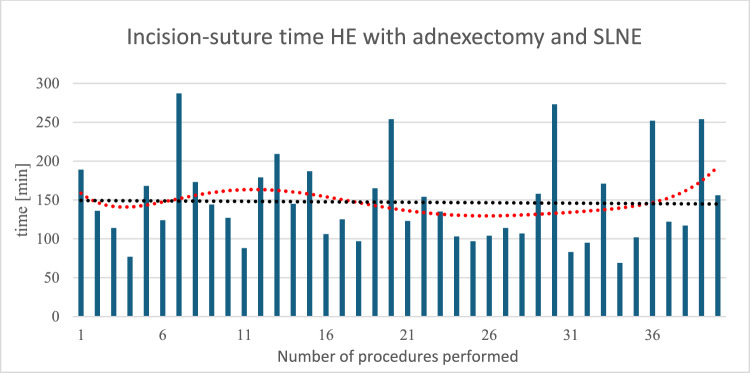
Shows skin-to-skin time during a hysterectomy with adnexectomy and SLNE. The trend line in red (degree 6 polynomial function) shows significant fluctuations in the values and does not indicate a reduction in operating times. The linear trend line in black shows a minimal decrease in skin-to-skin time over time

In the subgroup of patients with endometrial cancer who underwent a total hysterectomy with bilateral adnexectomy and sentinel lymph node dissection as the standard procedure, the mean skin-to-skin time was 147.08 min (± 55.05). The minimum and maximum skin-to-skin times for this procedure were 69 min and 287 min, respectively. The red trend line, which is as closely fitted to the values as possible, shows fluctuating dynamics. The complex dynamics of the red trend line do not reveal any obvious decrease or increase in skin-to-skin times. A minimal decrease in the mean skin-to-skin time can be seen in the simplified linear trend line in black. Figure 4 shows the skin-to-skin times when the standard procedure was performed on patients with endometrial cancer.

### Console time for malignant surgical indications

**Fig. 5:**
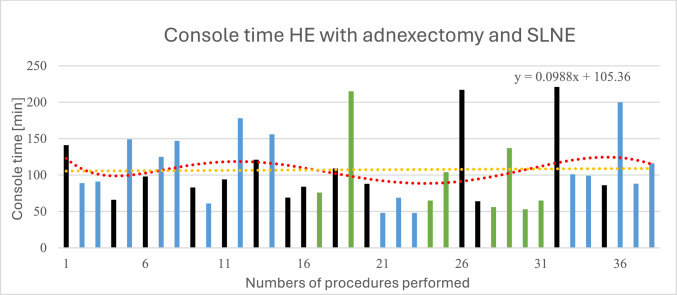
Console time during hysterectomy with adnexectomy and SLNE. The red trend line (polynomial grade 6) clearly shows fluctuations without a clearly discernible trend. The orange trend line (linear) does not show a clear increase or decrease in console time with an increasing number of procedures. Additionally, the individual bars are color-coded according to the surgeon: black = Surgeon A; blue = Surgeon B; green = Surgeon C

The average console time for patients with malignant surgical indications is 131.98 min (± 63.74). Since different surgical procedures were used for patients with cervical cancer and other malignant diseases such as ovarian cancer, depending on the stage, a more detailed analysis of these groups is omitted. The average console time for performing a hysterectomy with adnexectomy and SLNE is 109.89 min (± 52.04). The minimum and maximum console time durations are 48 min and 221 min, respectively. The color-coded bars allow for the assignment of individual values to the respective surgeon. The adjusted trend line in red shows a wave-like pattern with an initial decrease, followed by a rise and another decrease to a minimum, before rising again to higher values for console time. Overall, no clear trend in console times is discernible; the linear trend line in orange has an almost horizontal course (*y* = 0.0988x + 105.36). Figure 5 shows the console times for the operations on patients treated with hysterectomy with adnexectomy and SLNE for endometrial cancer.

### Learning curve

**Fig. 6:**
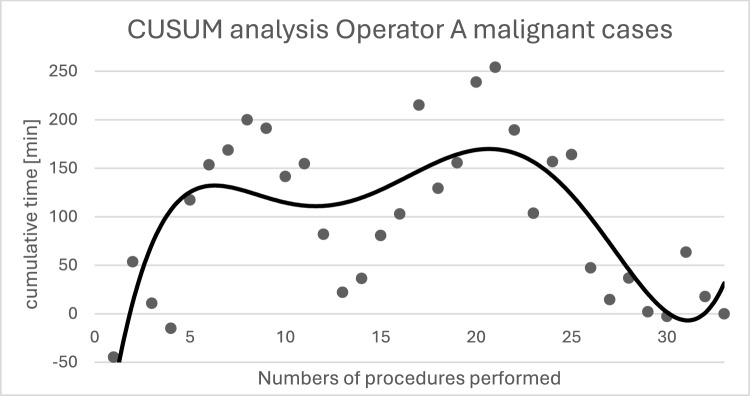
Shows the calculated CUSUM values for surgeon A during console time. The trend line is shown in black (6th-degree polynomial). The CUSUM peak occurs at approximately 11 procedures, and the second peak occurs at 28 procedures. After reaching the second peak, a steady decline in the CUSUM values can be observed

**Fig. 7:**
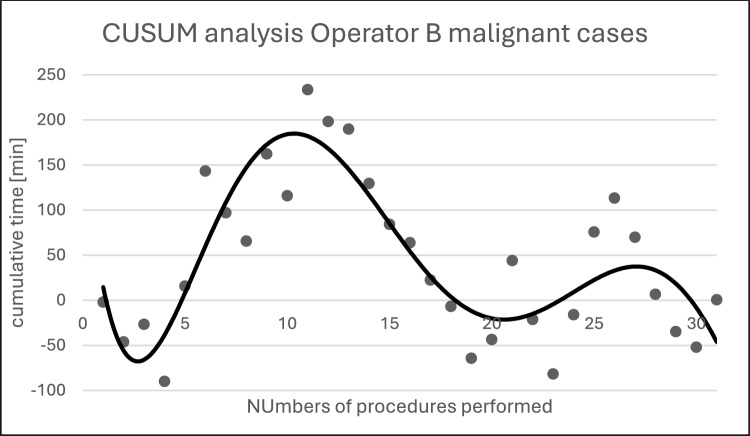
Shows the calculated CUSUM values for operator B during console time. The trend line, shown in black (6th degree polynomial), follows a bimodal curve. The CUSUM peak occurs at approximately 21 procedures. After reaching the peak, a significant decrease in the CUSUM values can be observed

Surgeon A performed a total of 42 procedures with an average console time of 133.76 min (± 58.74). The calculation of the CUSUM values is shown in Fig. 6 and exhibits a bimodal curve with a peak at approximately 11 procedures. After reaching the peak, a decrease in the CUSUM values is observed. Surgeon B performed a total of 40 robotic-assisted procedures with an average console time of 129.35 min (± 61.28). The results of the CUSUM analysis of Surgeon B's console time are shown in Fig. 7 and show a CUSUM peak after approximately 22 procedures. Surgeon C performed *n* = 24 procedures, and Surgeon D *n* = 1; these data are not shown.

In the entire cohort, the mean length of stay was 5.00 days (± 2.30). In the subgroup of patients with endometrial cancer, the median intraoperative blood loss was 50 ml (IQR 80 ml; Q1 20 ml, Q3 100 ml; *n* = 107). A total of two patients required conversion to open surgery, corresponding to a conversion rate of 1.9%. In one patient with endometrial cancer who had previously undergone surgery (1/107, 0.9%), severe bleeding of approximately 1000 ml occurred due to an injury to the right external iliac vein, necessitating conversion to open surgery. This conversion required vascular intervention. In the other patient, leads from a previously implanted gastric pacemaker obstructed the laparoscopic approach. For safety reasons, open surgery was therefore performed.

## Discussion

This retrospective analysis of the first *n* = 107 patients with gynecological malignancies who underwent surgery during the introductory phase of the da Vinci Surgical System at the Department of Gynecology, University Hospital Freiburg, provides valuable insights into the clinical application of robotic-assisted surgery in gynecologic oncology. The study examines numerous parameters, including patient-specific aspects, operating times, learning curves of individual surgeons and the surgical team, and complications. Unlike many studies, our dataset includes all gynecological malignancies (*n* = 107), which better reflect clinical reality.

In this cohort of first-time hysterectomies with adnexectomy and SLNE or LND for endometrial cancer, no significant reduction in intraoperative blood loss was observed with increasing surgical experience. This suggests that a stable level of safety regarding blood loss was achieved from the start of the robotic program.

Furthermore, the complexity of the procedure, including SLNE, LND and the presence of adhesions, did not significantly affect blood loss. These results suggest that robotic-assisted hysterectomy can be performed safely with respect to intraoperative bleeding. The absence of a measurable learning curve in blood loss may reflect the inherent precision and hemostatic advantages of the robotic surgical platform.

The study population essentially reflects the patient population encountered in everyday clinical practice. The inclusion criterion of "performing a robotic-assisted procedure" did not preselect patients from the overall cohort. Further categorization was only performed during the analysis of operating times to obtain more meaningful results. Selection based on other parameters, such as patient age or BMI, was deliberately omitted to reflect everyday clinical practice as realistically as possible.

This neglects potential further influences, such as the large age differences (23–83 years) among the patients, on surgical outcomes, particularly on operating times and complications. Regarding the distribution of surgical indications presented in the exploratory analyses, it should be noted that in the initial phase, significantly more patients with benign indications than those with malignant indications were deliberately operated on in order to establish a surgical routine in less complex cases. We have already published these data [7].

After the surgical team and individual surgeons had become proficient in using the robot in the operating room, an increasing number of patients with malignant surgical indications were operated on, which further increased the complexity of the procedures at that time.

For benign surgical indications, we were able to demonstrate a significant reduction in preparation time, skin-to-skin time, and console time within just a few procedures [9]. Analysis of preparation time shows an average of 28.1 min for the first 30 procedures, which is 4.4 min longer than for the subsequent 220 procedures. This confirms the surgical team's adaptation to the new workflows and is consistent with findings from other studies. Lenihan et al. [10] suggest in their study that a surgical team needs approximately 20 procedures to establish a routine for preparing the robot and to achieve a preparation time of less than 45 min, which improves further after 50 cases and decreases to approximately 35 min. Potential factors influencing preparation time, such as frequently changing operating room staff and prior experience with teamwork, lead to varying preparation times. Nevertheless, the phases of the learning process appear to be similar.

In our own cohort, we have already demonstrated that after an initial reduction in preparation time for benign surgical indications only, the average preparation time increased again when malignant surgical indications were introduced [9]. For patients with malignant surgical indications, the procedures vary, for example, due to the additional application of ICG during preparation. This different surgical preparation, as well as the additional measure, could explain an average 3-min longer preparation time in malignant cases compared with operations for benign surgical indications [9].

The average skin-to-skin time for patients undergoing benign hysterectomy in our cohort was 97.0 min, which was sometimes lower than those of previous studies [9, 11]. The skin-to-skin time for patients undergoing malignancy-related hysterectomy with adnexectomy and SLNE or LND was significantly higher, at 147.08 min, due to the greater complexity of the procedure, and it hardly decreased over time.

According to previous literature, reaching the learning curve was defined as requiring 75 operations per surgeon. We were able to demonstrate a significant reduction in skin-to-skin time within the first 30 procedures for benign hysterectomies [9]. We were unable to confirm this effect as clearly for malignant surgical indications in this study. This is likely due to the lower complexity of benign hysterectomies, which allows for the repetitive application of surgical techniques and thus faster learning.

In the time of the introduction of robotic-assisted surgery in the Department of Gynecology at the University Hospital of Freiburg, the surgeons had little prior experience in this area. While all surgeons were experienced in minimally invasive surgery, two of the four gynecologic oncologists had no prior experience, and the other two had limited experience with the da Vinci system. The average console time of these surgeons, at 95.5 min, is comparable to the results of previous studies. Rajanbabu et al., in their analysis with a similar timeframe and a comparable number of patients, found an average of approximately 103 min and observed a significant decrease in the average console time within the first year [12]. This decreased from an average of 130 min for the first 80 procedures in the first year of implementation to 95 min in the second year.

The individual console times of the different surgeons, as well as the evaluation of their learning curve using CUSUM analysis, show that console times decrease significantly after approximately 10–20 performances of the same procedure. Previous studies investigating learning curves yielded comparable results, describing the attainment of acceptable routine after about 20 procedures [8, 13–16].

The discernible differences in the presented learning curves may be attributed not only to prior surgical experience but also to the frequency of procedures performed during the professionalization process. A broad range of training and professional development opportunities is essential for the introduction of new technologies and their successful application in everyday clinical practice. For training in operating with the da Vinci Surgical System, both the manufacturer and our professional societies offer a comprehensive range of courses for surgeons and other healthcare professionals who use the da Vinci system (INTUITIVE SURGICAL OPERATIONS, INC., 2023e). To enable standardized training of the highest quality, the German Curriculum for Robotic Surgery in Gynecology (DCRG) was established by the German Society for Gynecological Endoscopy (AGE) (ARBEITSGEMEINSCHAFT ENDOSKOPIE, 2023).

In this study, the conversion rate for the first *n* = 107 evaluated operations in patients with malignant disease was 1.9%, which is consistent with other studies that describe similarly low conversion rates [17]. BORSE et al. observed a conversion rate of 1.4% in a study of 144 patients undergoing robotic-assisted hysterectomy [18]. A multicenter study with surgical data from 2300 patients undergoing robotic-assisted hysterectomy reported an even lower rate of only 0.1% with just two conversions [19]. The conversion rate to laparotomy in the Freiburg cohort, based on oncological cases, was 1.9%, significantly lower than the conversion rates reported in other studies with oncological cases during the initial implementation phase (typically 2–7%) [20, 21]. This indicates a highly effective integration process and good team adaptation.

The data show that robotic-assisted surgery is a very safe and reliable minimally invasive surgical option, even in complex patient groups. In early-stage endometrial cancer, robotic-assisted hysterectomy has become increasingly established as the preferred procedure, achieving comparable, if not better than, those of open or laparoscopic methods [22]. Robotic-assisted surgery is becoming increasingly established in the early stages of endometrial and cervical cancer. Its use in advanced ovarian cancer and other complex gynecological malignancies requires further research [21].

The learning curve of the Freiburg data on robotic-assisted gynecological oncology surgery shows a rapid increase in efficiency. The CUSUM analysis suggests that the team and surgeons achieved an increase in efficiency after approximately 11–22 procedures. This is at the lower end of the range reported in large European cohorts, where competence in terms of operating time and perioperative outcomes is typically achieved after 10–33 procedures [22, 23].

The advantages of the da Vinci Surgical System, such as the optimized view of the surgical field, the exceptional maneuverability of the instruments, and the ability to use optical support systems like Firefly mode or the TilePro function intraoperatively, assist the surgeon in performing the procedure safely. One limitation of our study is its retrospective analysis. Another relevant aspect arises from the heterogeneity of the study population, which necessitates further differentiation of the cohort. Particularly in patients with malignant surgical indications, despite further differentiation according to the type of procedure performed, the cohort remains highly heterogeneous due to individual tumor location and spread, as well as other factors. This heterogeneity inherent in the clinical, retrospective study design can lead to biases in the results, which must be considered, especially when interpreting the learning curves. To increase the comparability and significance of the learning curves, the comparison procedure of total hysterectomy with adnexectomy and sentinel lymph node dissection in endometrial cancer was selected, which on the one hand limits the transferability of the results to other procedures or indications and on the other hand results in the data series for the creation of the learning curves exhibiting a certain discontinuity. This is because maintaining the chronological order of the procedures meant that individual surgeons also performed benign operations in the interim, which are not included in the learning curve analysis. As a result, the time intervals between individual evaluated procedures vary considerably, and the experience gained through performing benign operations is also neglected, meaning that the resulting learning curves do not accurately reflect all learning effects over time. Furthermore, operators have significantly different case numbers, which is why the learning curves only allow for highly individual statements about the speed of learning. In this context, it should also be noted that while most current studies use CUSUM analysis to assess learning curves, this alone does not provide reliable information about when the learning curve has been reached. This is because the CUSUM peak is influenced by the total number of values included in the analysis. Since the average is calculated from the available data, the target value is therefore a self-reference. The number of cases in the CUSUM peak thus only indicates in which case the console time reaches the average value. Therefore, the number of cases determined using CUSUM peaks alone is not sufficient to assess whether the "learning curve has been overcome" [16].

The dataset published here is particularly valuable because it systematically examines both the individual learning curve of the surgeon and the learning curve of the team (preparation time) using CUSUM analysis. Most studies, in contrast, focus only on the operating times of individual surgeons [24]. To date, very little German data exist on the implementation of robotic-assisted gynecological oncology. Our study provides important data for the German-speaking region and the German healthcare system.

## Conclusion

In summary, this work provides new oncological data and valuable insights for the implementation of a robotic-assisted surgical program. The methodology is reliably applicable; for example, the conversion rate is very low. Reductions in individual operating times are observed after just a few procedures, typically around 20 per surgeon: preparation time, time from incision to suture closure, and console time. The individual console time values for each surgeon, as well as the evaluation of their learning curve using CUSUM analysis, show that a significant increase in efficiency is achieved after approximately 10–20 performances of the same procedure.

The introduction of robotic-assisted surgery in gynecological oncology is characterized by a steep learning curve, high patient safety, and successful integration into routine clinical practice.

## Data Availability

No datasets were generated or analysed during the current study.
